# A novel antisense lncRNA NT5E promotes progression by modulating the expression of SYNCRIP and predicts a poor prognosis in pancreatic cancer

**DOI:** 10.1111/jcmm.15718

**Published:** 2020-08-08

**Authors:** Pengbo Zhang, Meng Cao, Yi Zhang, Lei Xu, Fanchao Meng, Xinquan Wu, Tianfang Xia, Qun Chen, Guodong Shi, Pengfei Wu, Lei Chen, Zipeng Lu, Jie Yin, Baobao Cai, Shouji Cao, Yi Miao, Kuirong Jiang

**Affiliations:** ^1^ Pancreas Center The First Affiliated Hospital of Nanjing Medical University Nanjing China; ^2^ Department of General Surgery The Affiliated Hospital of Xuzhou Medical University Xuzhou China; ^3^ Department of General Surgery Nanjing Drum Tower Hospital The Affiliated Hospital of Nanjing University Medical School Nanjing China; ^4^ Department of Hepatopancreatobiliary Surgery The Third Affiliated Hospital of Soochow University Changzhou China; ^5^ Department of General Surgery The Affiliated Huaian No. 1 People’s Hospital of Nanjing Medical University Huai’an China; ^6^ Pancreas Institute Nanjing Medical University Nanjing China

**Keywords:** LncNT5E, long non‐coding RNAs, pancreatic cancer, prognosis, SYNCRIP

## Abstract

A novel antisense lncRNA NT5E was identified in a previous microarray that was clearly up‐regulated in pancreatic cancer (PC) tissues. However, its biological function remains unclear. Thus, we aimed to explore its function and clinical significance in PC. The lncNT5E expression was determined in PC specimens and cell lines. In vitro and in vivo studies detected the impact of lncNT5E depletion on PC cell proliferation, migration and invasion. Western blotting investigated the epithelial‐mesenchymal transition (EMT) markers. The interaction between lncNT5E and the promoter region of SYNCRIP was detected by dual‐luciferase reporter assay. The role of lncNT5E in modulating SYNCRIP was investigated in vitro. Our results showed that lncNT5E was significantly up‐regulated in PC tissues and cell lines and associated with poor prognosis. LncNT5E depletion inhibited PC cell proliferation, migration, invasion and EMT in vitro and caused tumorigenesis arrest in vivo. Furthermore, SYNCRIP knockdown had effects similar to those of lncNT5E depletion. A significant positive relationship was observed between lncNT5E and SYNCRIP. Moreover, the dual‐luciferase reporter assays indicated that lncNT5E depletion significantly inhibited SYNCRIP promoter activity. Importantly, the malignant phenotypes of lncNT5E depletion were rescued by overexpressing SYNCRIP. In conclusion, lncNT5E predicts poor prognosis and promotes PC progression by modulating SYNCRIP expression.

## 1 INTRODUCTION

Pancreatic cancer (PC) is one of the most aggressive and leading lethal malignancies and has a very poor prognosis.^1^ It is the seventh leading cause of cancer‐related death in both men and women globally.^2^ In the United States, PC is the fourth leading cause of cancer‐related death^3^ and is projected to rise to become the second leading cause within the next decade.^4^ Despite advances in molecular mechanism research and therapeutic approaches for PC during the past few decades, the 5‐year survival rate of approximately 9% remains dismal, the lowest rate among any malignant tumours.^3^ This extremely poor prognosis is mainly influenced by extensive local invasion and early distant metastasis. Radical surgery in combination with chemotherapy and radiotherapy is the only potentially curative treatment strategy, but fewer than 20% of patients are eligible for resection^1^ because of rapid progression and late diagnosis. Thus, it is urgent to explore the underlying molecular mechanisms of PC tumorigenesis and progression and identify innovative diagnostic biomarkers and therapeutic targets for PC.

Long non‐coding RNAs (lncRNAs), which are defined as transcripts of more than 200 nucleotides with no or limited protein‐coding function,^5^, ^6^ have attracted increasing attention because of their diverse biological functions in development and diseases,^7^, ^8^, ^9^ particularly carcinogenesis and progression of cancers.^10^, ^11^, ^12^, ^13^, ^14^, ^15^, ^16^ Aberrant expression of lncRNAs has been demonstrated in various types of cancers, such as lung cancer,^17^ colorectal cancer,^18^ prostate cancer,^19^ gastric cancer,^20^ breast cancer,^21^ liver cancer^22^ and pancreatic cancer.^23^, ^24^ Multiple lncRNAs are associated with cancer cellular oncogenesis, proliferation, migration, invasion, differentiation and metastasis. Thus, lncRNAs are likely to represent a new class of potential diagnostic biomarkers and therapeutic targets for cancers. Within these lncRNAs, a particular group are natural antisense transcripts from the opposite strand of protein‐coding transcripts, termed antisense lncRNAs. Antisense lncRNAs, located either in the nucleus or in the cytoplasm, are functionally diverse. They can modify the expression of neighbouring genes in cis or more distant genes in trans through various mechanisms to exert a wide variety of biological functions.^25^ In our previous study, we performed microarrays to explore the expression profiles of lncRNAs and associated mRNAs in PC. A large number of differentially expressed lncRNAs and mRNAs were identified between PC and adjacent normal tissues, among which three markedly down‐regulated lncRNAs (lncPCTST, XLOC_000647 and KCNK15‐AS1) in PC tissues were reported to be correlated with the progression and prognosis of PC.^26^, ^27^, ^28^ A novel antisense lncRNA NT5E (ENST00000421594), identified in microarrays, was also significantly up‐regulated (fold change: 10.78) in PC tissues. LncNT5E, located on human chromosome 6q14.3, is a broadly expressed and evolutionarily non‐conservative lncRNA with a length of 554 base pairs (bp). However, the biological function and clinical value in PC progression have not been fully elucidated so far.

In the current study, we evaluated the expression levels of lncNT5E in PC tissues and cell lines and investigated its clinical significance. Then, we assessed the biological functions of lncNT5E on PC cell oncogenic phenotypes, including cell proliferation, migration, invasion and epithelial‐mesenchymal transition (EMT), both in vitro and in vivo. Finally, we explored the potential molecular mechanism of lncNT5E in PC progression. Thus, this study sheds light on lncNT5E as a promising potential novel molecular biomarker and therapeutic target for PC.

## 2 MATERIALS AND METHODS

### 2.1 Patients and clinical samples

A total of 45 paired PC and matched adjacent normal tissue specimens were collected from patients who underwent pancreatic resection at the Pancreas Center, the First Affiliated Hospital of Nanjing Medical University (Nanjing, China), from February 2016 to February 2017. The protocol was approved by the Hospital Ethics Committee, and written informed consent was signed from all patients before surgery. No patients had distant metastasis or received anticancer treatment before surgery. All specimens were evaluated and confirmed to be pancreatic ductal adenocarcinoma (PDAC) by a certified pathologist. All samples were snap‐frozen in liquid nitrogen and then stored at −80°C until required. Complete follow‐up data were acquired. Overall survival (OS) was defined as the time from the date of surgery to the date of death or the end of follow‐up (November 2019), and progression‐free survival (PFS) was the time interval between the date of surgery and the date of objective tumour progression or death or the end of follow‐up.

### 2.2 Cell lines and cell culture

Human PC cell lines (CFPAC, COLO‐357, PANC‐1 and BXPC‐3) and human embryonic kidney cells (293T) were procured from the American Type Culture Collection (ATCC). The normal human pancreatic duct cell line HPNE was obtained from Pancreas Institute, Nanjing Medical University. All cells were cultured at 37°C in a humidified atmosphere with a 5% CO2 incubator and maintained in Dulbecco's modified Eagle's medium (DMEM; KeyGen BioTECH) or RPMI‐1640 medium (Gibco) supplemented with 10% foetal bovine serum (FBS, Gibco), 100 U/mL penicillin and 100 mg/mL streptomycin (Gibco).

### 2.3 Small interfering RNA (siRNA) transfection and plasmid infection

According to the manufacturer's protocol, when PC cells grew to 30%~50% confluency, siRNA specific for lncNT5E (siLncNT5E) and non‐specific control (si‐ctrl) (GenePharma) were transfected with siLentFect Lipid Reagent (Bio‐Rad). After transfection for 6 hours, fresh medium was used to replace the medium containing transfection reagents. The SYNCRIP‐overexpressing plasmid was purchased from GenePharma. Briefly, SYNCRIP cDNA was cloned to the pcDNA3.1 vector. Prior to plasmid transfection, PC cells grew to 80%~90% confluency, and then, the pcDNA3.1 plasmid with full‐length lncNT5E (GenePharma) or the empty vector as a control was transfected with the aid of Lipofectamine 2000 (Invitrogen). The transfection lasted for 6 hours. The transfection effect was verified by qRT‐PCR. Forty‐eight hours after, the treated cells were used for various cell assays. The siRNA sequences were as follows:

For lncNT5E, siRNA#1:5′‐CCAGCAACAUACCACACAATT‐3′;

siRNA#2:5′‐CCAACACCUACUACAUAAUTT‐3′;

siRNA#3:5′‐GGGUGGUAAGGUGCUCAAUTT‐3′.

For SYNCRIP, siRNA#1:5′‐GGGAAAGAUCCCAAGAGAUTT‐3′;

siRNA#2:5′‐GCAUCUCAGUUGCCAACAATT‐3′;

siRNA#3:5′‐GCUGUUUGUACGCAACCUUTT‐3′.

### 2.4 Construction of the lncNT5E stable expression cell line

A lentiviral pGLV2‐U6‐Puro vector containing lncNT5E shRNA and a negative control lentivirus were purchased from GenePharma (GenePharma). They were cotransfected into 293T cells using EndoFectin Lenti transfection reagent according to the manufacturer's instructions. After culturing for 48 hours, the lentiviral particles in the supernatant were harvested, filtered and then used to transfect CFPAC cells. To select stably transduced cells, the cells were resuspended and cultured in 2 µg/mL puromycin (Meilunbio) for 2 weeks; qRT‐PCR was performed to determine the level of lncNT5E expression.

### 2.5 RNA extraction and quantitative real‐time PCR (qRT‐PCR)

Total RNA samples from cells and tissue specimens were extracted using TRIzol Reagent (Invitrogen) following the manufacturer's instructions and then reverse‐transcribed into complementary DNA (cDNA) using HiScript Q RT SuperMix for qPCR (+gDNA wiper) (Vazyme). The amplification reaction volume was 20 µL containing 10 µL SYBR Green PCR Master Mix (Vazyme), 2 µL cDNA, 7.2 µL H_2_O and 0.8 µL amplification primers. qRT‐PCR was performed on a LightCycler^®^ 480 Real‐Time PCR Instrument (Roche). 18S was used as a standard internal reference. All samples were analysed in triplicate. The results were normalized and analysed using the fold change (2^−ΔΔCT^) method. The qRT‐PCR primer sequences were as follows:

lncNT5E‐Primer: forward: 5′‐GAATTGCAGCATCACCAGCCTTG‐3′;

reverse: 5′‐CTGACTTGCTGACCAGTGCTCTC‐3′.

SYNCRIP‐Primer: forward: 5′‐CTGGTCTCAATAGAGGTTATGCG‐3′;

reverse: 5′‐TCCGGTTGGTGGTATAAAATGAC‐3′.

18S‐Primer: forward: 5′‐GTAACCCGTTGAACCCCATT‐3′;

reverse: 5′‐CCATCCAATCGGTAGTAGCG‐3′.

### 2.6 Western blotting

Tissues or cells were homogenized and lysed with RIPA lysis buffer. After determining the protein concentration using a BCA assay (Thermo Fisher Scientific), equal amounts of proteins were separated via sodium dodecyl sulphate‐polyacrylamide gel electrophoresis (SDS‐PAGE; 10% gel) and then transferred onto a polyvinylidene fluoride (PVDF) membrane (Millipore). The membranes were blocked with 5% skim milk for 1 hour at room temperature and incubated with specific primary antibodies overnight at 4°C with gentle shaking. After incubation in a secondary antibody for 2 hours at room temperature, the immunoreactive bands were visualized by an enhanced chemiluminescence system (SuperSignal West Femto trial kit, Pierce), and the band intensities were quantified using an image analysis system. Primary antibodies were as follows: E‐cadherin (1:300, CST); vimentin (1:1000, CST); N‐cadherin (1:300, CST); SYNCRIP (1:10 000, ab184946, Abcam); cleaved caspase‐3, cleaved caspase‐7 and cleaved caspase‐9 (1:2000, Proteintech); and GAPDH (1:20 000, CST).

### 2.7 Cell proliferation assay

Cell proliferation was measured using Cell Counting Kit‐8 (CCK‐8, Dojindo) and EdU staining assay following the manufacturer's instructions. In CCK‐8 assay, after transfection for 48 hours, cells were seeded in 96‐well plates at a density of 5 × 10^3^ cells per well. The absorbance at 450 nm was measured with a Cytation 5 microplate spectrophotometer (BioTek) following incubation for 2 hours at 37°C after adding 10 µL CCK‐8 solution to each well. In EdU staining assay, after transfection for 48 hours, cells were seeded in 96‐well plates at a density of 2 × 10^4^ cells per well. Cells incubate for 2 hours at 37°C after adding diluted EdU solution at ratio of 1:1000 with medium containing foetal bovine serum and then fixed with paraformaldehyde. The 100 µL 1× Apollo^®^ dyeing reaction solution and 100 µL 1× Hoechst 33 342 reaction solution were added to each well and incubated for 30 minutes, respectively. Finally, the DNA was stained with Hoechst 33 342, and the fluorescence was measured at 594 and 350 nm with fluorescence microscope (Olympus). The positive proportion of nucleated cells for EdU was evaluated. The experiments were repeated three times.

### 2.8 Cell migration and invasion assays

Cell migration and invasion ability were evaluated using Transwell chambers (24‐well insert, 8 µm pore size, Millipore). A total of 5 × 10^4^ cells were seeded in the upper chamber with 200 µL serum‐free medium, and the bottom chamber was filled with 800 µL culture medium supplemented with 20% foetal bovine serum (FBS) as a chemoattractant to induce cell migration. After incubation for 48 hours, non‐migrated cells were removed, and cells permeating the chamber membrane were fixed with paraformaldehyde and then stained with 0.1% crystal violet. The number of migratory cells was counted under a microscope in five random fields. The cell invasion assay was performed using Matrigel‐coated Transwell chambers. Other procedures were similar to the migration assay.

### 2.9 Dual‐luciferase reporter assay

The promoter region of SYNCRIP (from −1391 to 138) was chosen to design primer and then amplified by PCR and inserted into the pGL3‐Basic vector (GenePharma Company). In this study, luciferase reporter plasmids included SYNCRIP promoter‐pGL3‐Basic (referred to as pGL3‐promoter) and pGL3‐Basic (negative control). 293T cells (1 × 10^4^ per well) were inoculated in 48‐well plates with 200 µL culture medium and then cotransfected with the target siRNA and plasmid (si‐ctrl or si‐LncNT5E and pGL3‐Basic or pGL3‐promoter) and pRL‐TK plasmid expressing Renilla luciferase (Promega). After incubation for 48 hours, cells were harvested, and luciferase activity was measured with a Dual‐Luciferase Reporter Assay System (Promega). Renilla luciferase activity was used for normalization. The luciferase activity was expressed in terms of the ratio of firefly luciferase activity to Renilla luciferase activity.

### 2.10 Tumour formation assay in vivo

Briefly, 6‐week‐old female athymic nude mice (BALB/c) were purchased from Vital River Laboratory Animal Technology Co. A total of 5 × 10^6^ CFPAC cells stably expressing sh‐ctrl or shLncNT5E were resuspended in 200 µL medium (100 µL PBS + 100 µL Matrigel) and then subcutaneously inoculated into the flank of each nude mouse. The tumour size was measured with a calliper every 2 days, and the tumour volume was calculated using the following formula: length × (width)^2^/2. The weight of xenograft tumours was measured when harvested 18 days after inoculation. The harvested samples were fixed in 4% formaldehyde, embedded in paraffin, sectioned (5 µm) and subjected to haematoxylin and eosin (HE) and immunohistochemical (IHC) staining.

All animal experiments were approved by the Animal Care and Use Ethics Committee of Nanjing Medical University and conducted following the ARRIVE (Animal Research: Reporting In Vivo Experiments) guidelines.

### 2.11 Immunohistochemical (IHC) staining

SYNCRIP staining was examined in human PC tissue specimens, and Ki67 staining was examined in xenograft tumour specimens from nude mice. Formalin‐fixed tissues were embedded in paraffin. The 5‐µm tissue sections were deparaffinized in xylene, rehydrated in a series of graded alcohols and rinsed in distilled water. Following antigen retrieval in citrate buffer (pH 6.0) for 25 minutes and endogenous peroxidases blocked in 3% H_2_O_2_ for 15 minutes, the slides were incubated with primary antibodies at 4°C overnight. The staining was then revealed via a horseradish peroxidase‐conjugated secondary antibody and 3,3′‐diaminobenzidine (DAB). Primary antibodies included SYNCRIP (1:100, ab184946, Abcam) and Ki67 (1:500, ab92742, Abcam).

The staining score for PC cells and adjacent normal cells was analysed separately, and multiple regions were recorded. The percentage of immunostaining and the staining intensity (0, negative; 1+, weak; 2+, moderate; and 3+, strong) were assessed. A histochemistry score (H‐score) ranging from 0 to 300 was recorded using the following formula^29^:

H‐score = (% of cells of weak intensity × 1) + (percentage of cells of moderate intensity × 2) + (percentage of cells of strong intensity × 3).

### 2.12 Statistical analysis

All values are presented as the mean ± standard deviation (SD). The quantitative data were analysed by Student's *t* test or one‐way ANOVA. The categorical variables were verified by Pearson's chi‐squared test (*χ* ^2^ test) or Fisher's exact test. Survival analysis was assessed using the Kaplan‐Meier method and analysed by the log‐rank test. Univariate and multivariate Cox regression analyses were performed to evaluate the relative risk factors for prognosis. Correlation analysis was verified by Pearson's correlation. Data analyses and graph presentation were performed using SPSS version 20.0 (SPSS, Inc) and GraphPad Prism 7.0 (GraphPad Software). *P* < .05 was considered statistically significant.

## 3 RESULTS

### 3.1 LncNT5E is aberrantly up‐regulated in PC tissues and cell lines

**FIGURE 1:**
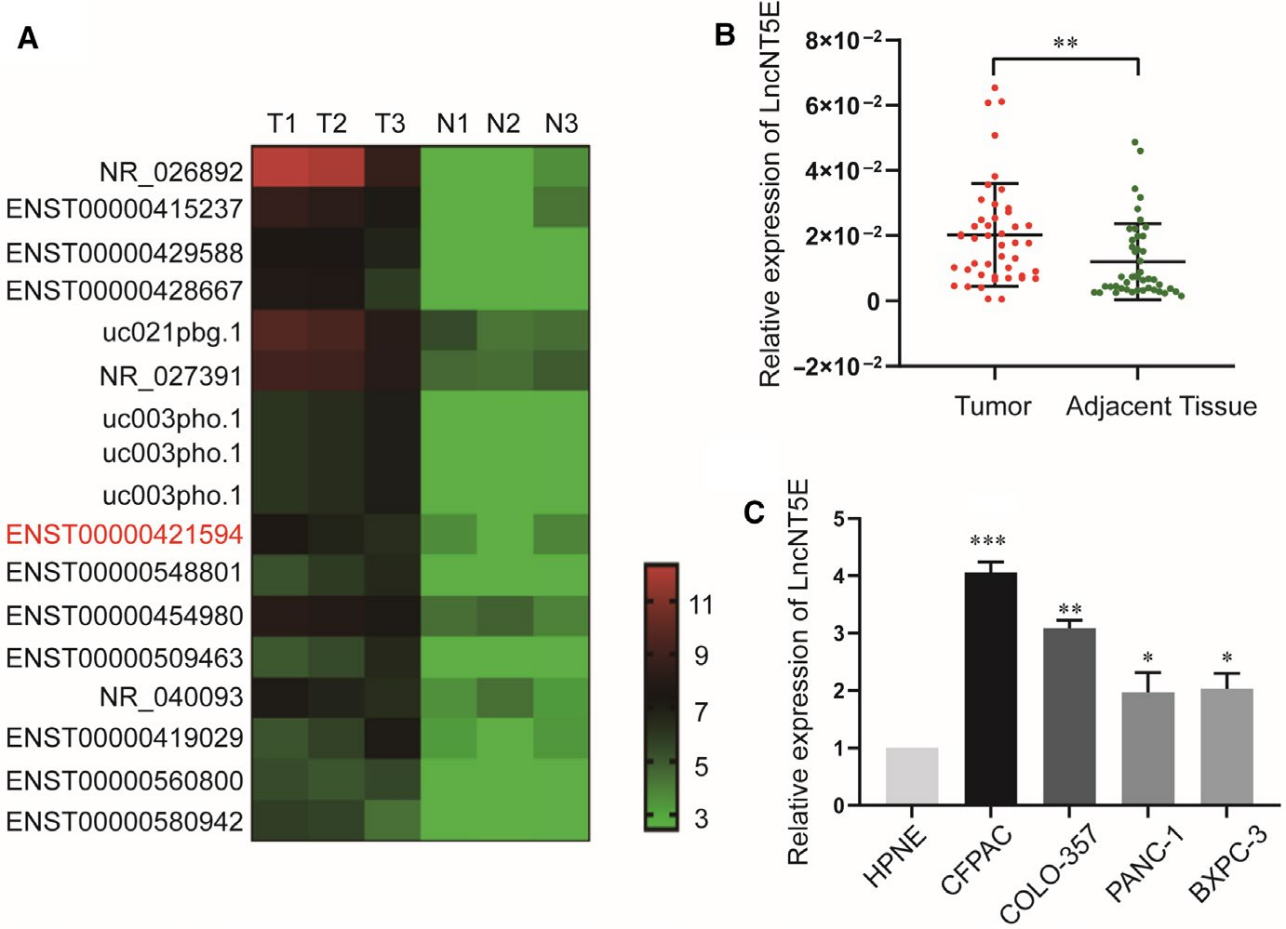
LncNT5E is up‐regulated in pancreatic cancer (PC) tissues and cell lines. A, The heat map from our previous lncRNA microarray reflected the differentially expressed lncRNAs in PC and normal tissues. T represents PC tissue, and N represents normal pancreatic tissue. ENST00000421594 indicates lncNT5E. B, Relative expression of lncNT5E in 45 paired PC and adjacent normal tissues. LncNT5E expression from all tissues was normalized to 18S expression (ΔCT) and then compared with adjacent normal tissues and converted to the fold change (2^−ΔΔCT^). C, Relative expression of lncNT5E in different cell lines. Data are shown as fold change (2^−ΔΔCT^). *P < .05, **P < .01, ***P < .001

We used microarray detection (H1602063, Arraystar Human LncRNA 8 × 60 k v3.0 1‐color) to study lncRNAs in three pairs of PC and adjacent normal tissues. We identified 17 up‐regulated lncRNAs in PC tissues (fold change > 2, *P* < .05, and a false discovery rate < 0.05) by microarray (Figure 1A) and then validated these results in PC tissues and cell lines. The qRT‐PCR results indicated the lncRNA NT5E (ENST00000421594) had good repeatability and specificity (fold change = 10.78, *P* = .014). Thus, we chose lncNT5E for further analysis. LncNT5E is mapped to human chromosome 6, is 554 bp in length and is an antisense lncRNA (https://lncipedia.org).

In this study, we further examined the expression of lncNT5E in 45 pairs of PC and adjacent normal tissues by qRT‐PCR. Compared with normal tissues, lncNT5E was markedly increased in PC tissues (Figure 1B). Then, we validated that the expression level of lncNT5E in four PC cell lines (CFPAC, COLO‐357, PANC‐1 and BXPC‐3) was dramatically higher in PC cell lines, especially CFPAC and COLO‐357, than in the normal human pancreatic ductal epithelial cell line HPNE (Figure 1C).

### 3.2 LncNT5E is associated with the clinicopathological features and prognosis of PC patients

**FIGURE 2:**
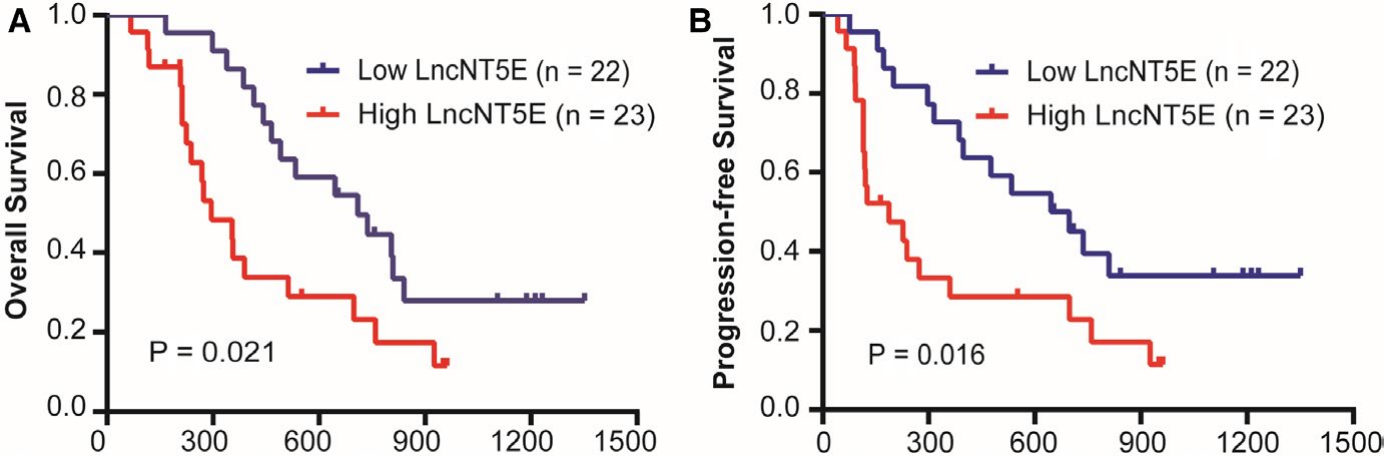
LncRNA is associated with poor prognosis of PC patients. Kaplan‐Meier curves of overall survival (A) and progression‐free survival (B) based on high or low expression levels of lncNT5E. The *P*‐values are shown by log‐rank test (two‐side).*P < .05

We investigated the clinical significance of lncNT5E in PC patients. As shown in Table 1, the results indicated that high expression of lncNT5E was significantly correlated with advanced TNM stage and lymph node metastasis. In addition, Kaplan‐Meier survival analysis revealed that PC patients with high lncNT5E expression had significantly shorter OS (log‐rank test, *P* = .021, Figure 2A) and PFS (log‐rank test, *P* = .016, Figure 2B) than patients with low expression. Moreover, univariate analysis demonstrated that high lncNT5E expression, perineural invasion, advanced TNM stage and advanced N stage were significantly associated with an increased risk of cancer recurrence and cancer‐related death (Table 2). Further multivariate analysis revealed that high lncNT5E expression and perineural invasion were independent prognostic factors indicating a poor prognosis in PC patients (Table 3). These results indicate that lncNT5E plays an oncogenic role in PC.

**Table 1:** Association between lncNT5E expression and clinicopathological parameters of PC patients

Characteristics	No of cases	lncNT5E level		*χ* ^2^	*P*‐value
		High	Low		
Total cases	45	23	22		
Gender^a^					
Male	33	19	14	2.070	.150
Female	12	4	8		
Age (years)^a^					
>60	30	15	15	0.044	.833
≦60	15	8	7		
Tumour location^a^					
Head	28	16	12	1.079	.299
Body and tail	17	7	10		
Differentiation^b^					
High	2	0	2	2.961	.228
Moderate	13	7	6		
Low	30	16	14		
Perineural invasion^a^					
Yes	33	18	15	0.584	.445
No	12	5	7		
TNM stage (AJCC)^b^, ^c^					
I	18	5	13	6.719	.035*
II	24	16	8		
III	3	2	1		
T stage^b^					
T1	11	4	7	2.709	.258
T2	26	16	10		
T3	8	3	5		
Lymph node metastasis^b^					
N0	21	6	15	9.195	.010**
N1	20	15	5		
N2	4	2	2		

Abbreviations: No of cases, number of cases; T stage, tumour stage; TNM, tumour‐node‐metastasis.

^a^ Pearson chi‐squared test.

^b^ Fisher's exact test.

^c^ American Joint Committee on Cancer (AJCC), patients were staged in accordance with the 8th Edition of the AJCC Cancer TNM Classification.

* *P* < .05.

** *P* < .01.

**Table 2:** Univariate and multivariate analysis of overall survival in PC patients (n = 45)

Variables	Univariate analysis		Multivariate analysis	
	HR (95% CI)	*P* value	HR (95% CI)	*P* value
Gender	0.804 (0.372‐1.734)	.578		
Male				
Female				
Age (years)	0.826 (0.393‐1.737)	.614		
≦60				
>60				
Tumour location	0.574 (0.275‐1.198)	.139		
Head				
Body and tail				
Differentiation	0.727 (0.402‐1.316)	.293		
Low				
High				
Moderate				
Perineural invasion	0.229 (0.087‐0.607)	.003**	0.288 (0.103‐0.807)	.009**
Yes				
No				
TNM stage (AJCC)	2.798 (1.513‐5.174)	.001***	1.490 (0.496‐4.478)	.478
I				
II				
III				
T stage	1.397 (0.886‐2.202)	.150		
T1				
T2				
T3				
N stage	2.063 (1.236‐3.445)	.006**	1.241 (0.515‐2.993)	.630
N0				
N1				
N2				
LncNT5E expression	2.214 (1.107‐4.426)	.025*	2.100 (1.025‐4.3001)	.043*
Low				
High				

Note

Cox regression analysis, *P < .05, **P < .01, ***P < .001.

Abbreviations: 95% CI, 95% confidence interval; AJCC, American Joint Committee on Cancer; HR, hazard ratio.

**Table 3:** Univariate and multivariate analysis of progression‐free survival in PC patients (n = 45)

Variables	Univariate analysis		Multivariate analysis	
	HR (95% CI)	*P* value	HR (95% CI)	*P* value
Gender	0.570 (0.246‐1.320)	.190		
Age (years)	0.780 (0.371‐1.641)	.513		
Tumour location	0.779 (0.384‐1.578)	.488		
Differentiation	0.628 (0.329‐1.202)	.160		
Low				
High				
Moderate				
Perineural invasion	0.339 (0.137‐0.835)	.019*	0.422 (0.160‐1.110)	.080
Yes				
No				
TNM stage (AJCC)	2.400 (1.322‐4.357)	.004**	1.439 (0.478‐4.338)	.518
I				
II				
III				
T stage	1.339 (0.847‐2.115)	.211		
T1				
T2				
T3				
N stage	1.872 (1.131‐3.098)	.015*	1.218 (0.500‐2.966)	.663
N0				
N1				
N2				
LncNT5E expression	2.306 (1.147‐4.638)	.019*	2.172 (1.056‐4.468)	.035*
Low				
High				

Note

Cox regression analysis, * *P* < .05, **P < .01.

Abbreviations: 95% CI, 95% confidence interval; AJCC, American Joint Committee on Cancer; HR, hazard ratio.

### 3.3 LncNT5E promotes the proliferation and EMT of pancreatic cancer cells

**FIGURE 3:**
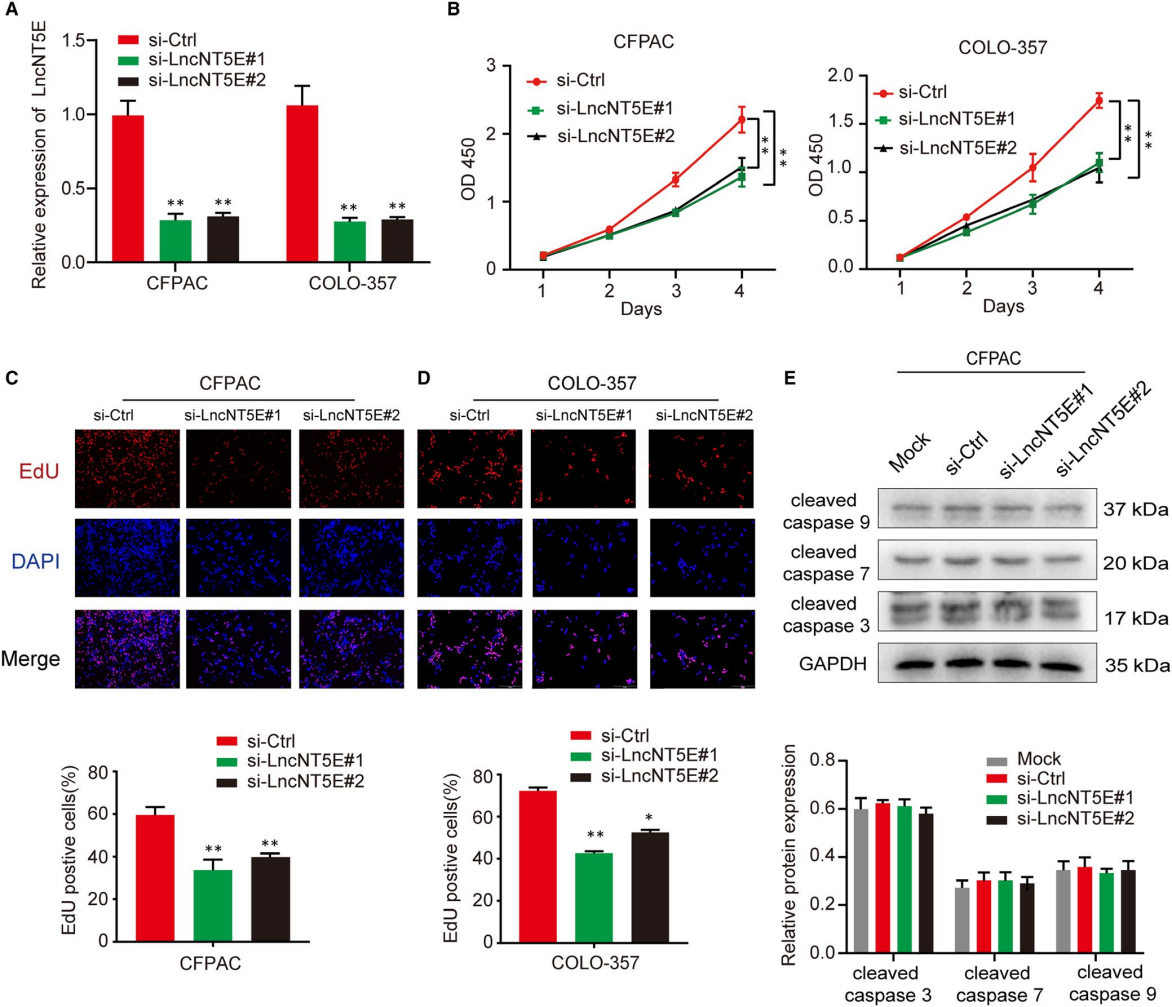
LncNT5E promotes PC cell proliferation in vitro. A, The expression of lncNT5E was down‐regulated in CFPAC and COLO‐357 cells transfected with lncNT5E siRNA (si‐lncNT5E#1 or si‐lncNT5E#2). B‐D, The proliferation activity of the CFPAC and COLO‐357 cells transfected with lncNT5E siRNA determined by CCK‐8 (B) and EdU staining (C and D) assays. E, The expression of mock and apoptosis‐related proteins (cleaved caspase‐3, cleaved caspase‐7 and cleaved caspase‐9) in CFPAC transfected with lncNT5E siRNA detected by Western blotting. Results are represented as protein intensity relative to GAPDH.*P < .05, **P < .01

We examined the potential role of lncNT5E in regulating PC progression in vitro. The lncNT5E siRNA was transfected into CFPAC and COLO‐357 cell lines, and the transfection efficiency was confirmed by qRT‐PCR (Figure 3A). The CCK‐8 assay demonstrated that lncNT5E depletion in two PC cell lines resulted in significant tumour growth inhibition (Figure 3B). Furthermore, we performed EdU staining assay to confirm that lncNT5E depletion down‐regulated the proliferation of the PC cells. The results showed that the positive proportion of EdU staining nuclei was significantly decreased in si‐LncNT5E group compared to that in si‐ctrl group (Figure 3C and D). Moreover, we investigated the expression of apoptosis‐related proteins (cleaved caspase‐3, cleaved caspase‐7 and cleaved caspase‐9) via Western blotting. The results showed that the expression levels of cleaved caspase‐3, cleaved caspase‐7 and cleaved caspase‐9 were not different under silencing of lncNT5E (Figure 3E). These results indicated that lncNT5E depletion really inhibited proliferation rather than increased cell death.

**FIGURE 4:**
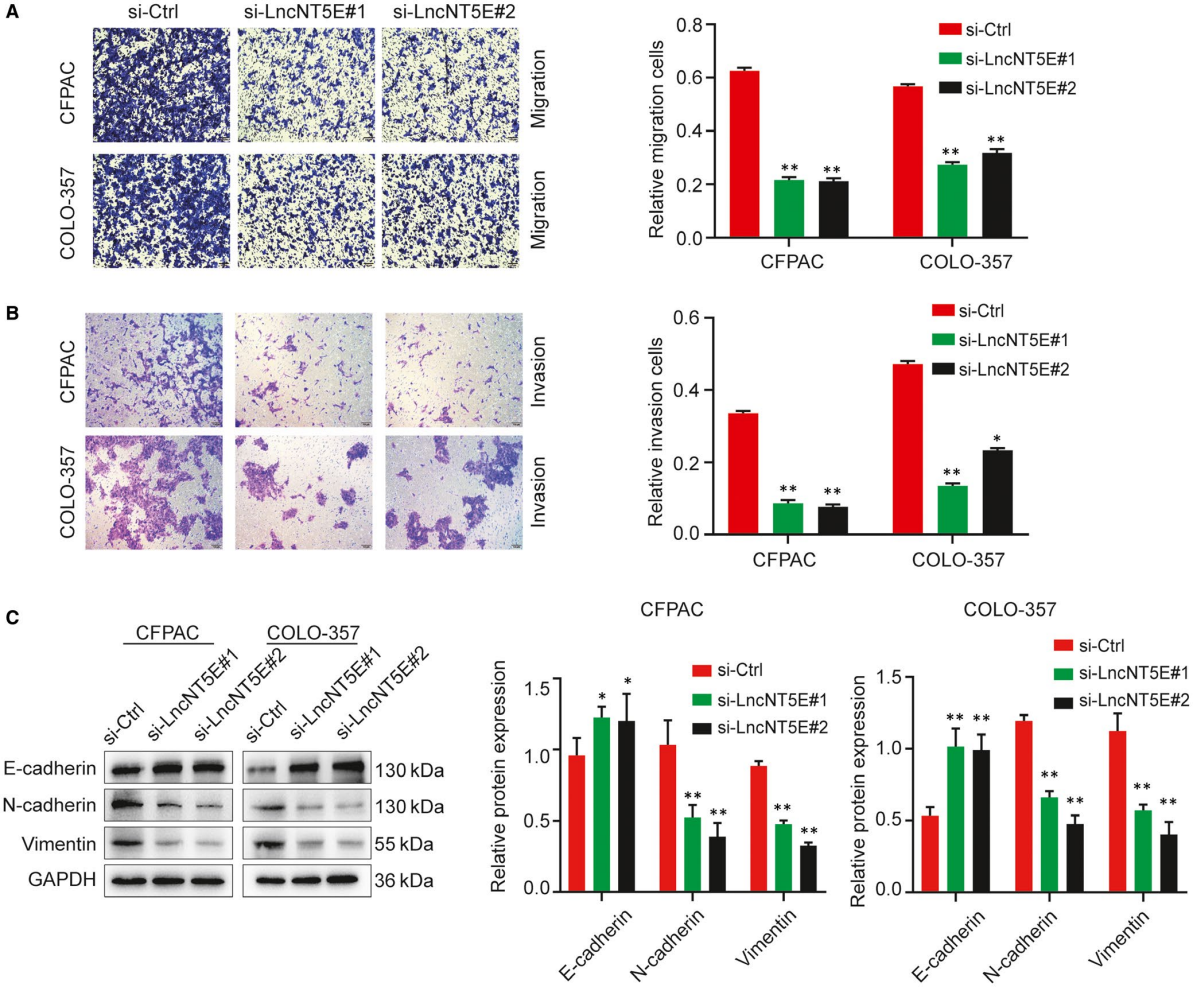
LncNT5E promotes PC cells migration, invasion and EMT in vitro. A and B, The migration (A) and invasion (B) capacity of the CFPAC and COLO‐357 cells transfected with lncNT5E siRNA by Transwell assays. C, Analysis of the E‐cad, N‐cad and vimentin protein levels in the CFPAC and COLO‐357 cells (si‐lncNT5E#1 or si‐lncNT5E#2 and si‐ctrl), as detected by Western blotting. Results are represented as protein intensity relative to GAPDH.*P < .05, **P < .01

In addition, the migration and invasion capacities were evaluated by Transwell assays. The results showed that invasion was inhibited in CFPAC and COLO‐357 cells with lncNT5E depletion (Figure 4A and B). We also detected the expression of several EMT markers via Western blotting. LncNT5E depletion led to increased levels of the epithelial marker E‐cadherin and decreased levels of the mesenchymal markers vimentin and N‐cadherin (Figure 4C). Therefore, these data suggest that lncNT5E can promote the proliferation and EMT of PC cells.

### 3.4 LncNT5E depletion inhibits tumour growth in vivo

**FIGURE 5:**
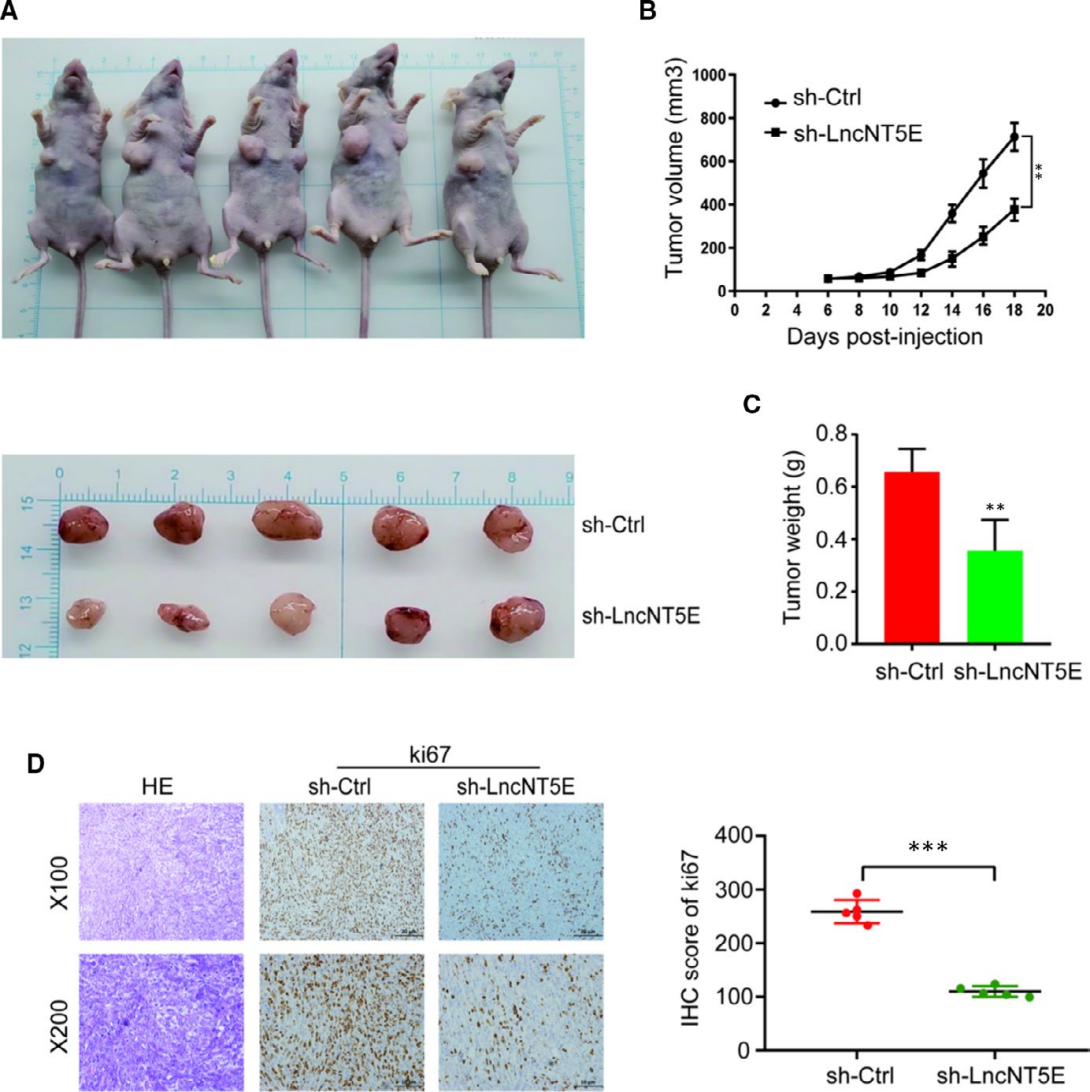
Stable lncNT5E depletion significantly inhibits tumour growth in vivo. A, Nude mice were killed 18 d after subcutaneous injection of CFPAC cells stably transfected with sh‐lncNT5E or sh‐ctrl, and tumour nodules were exhibited. B, Tumour volume curve of the sh‐lncNT5E or sh‐ctrl treatment groups was measured and analysed every 2 d. C, Tumour weights of sh‐lncNT5E or sh‐ctrl groups were measured when the tumours were collected. D, HE (haematoxylin and eosin) and IHC (immunohistochemical) staining of sections from subcutaneous xenograft tumours (×100 and ×200). The H‐score showed that stable lncNT5E depletion decreased the proliferation index of Ki67. Data are shown as the mean ± SD. **P < .01, ***P < .001

To further determine the function of lncNT5E in vivo, CFPAC cells with stable lncNT5E knockdown were subcutaneously injected into nude mice. The volume and weight of xenograft tumours in the stable lncNT5E knockdown group were markedly smaller than those in the control group (Figure 5A‐C). Moreover, immunohistochemical staining showed that the cell proliferation indicator Ki67 of tumours was decreased in the stable lncNT5E knockdown group (Figure 5D). These results suggest that lncNT5E promotes the tumorigenicity of PC cells in vivo.

### 3.5 LncNT5E promotes transcription of SYNCRIP

**FIGURE 6:**
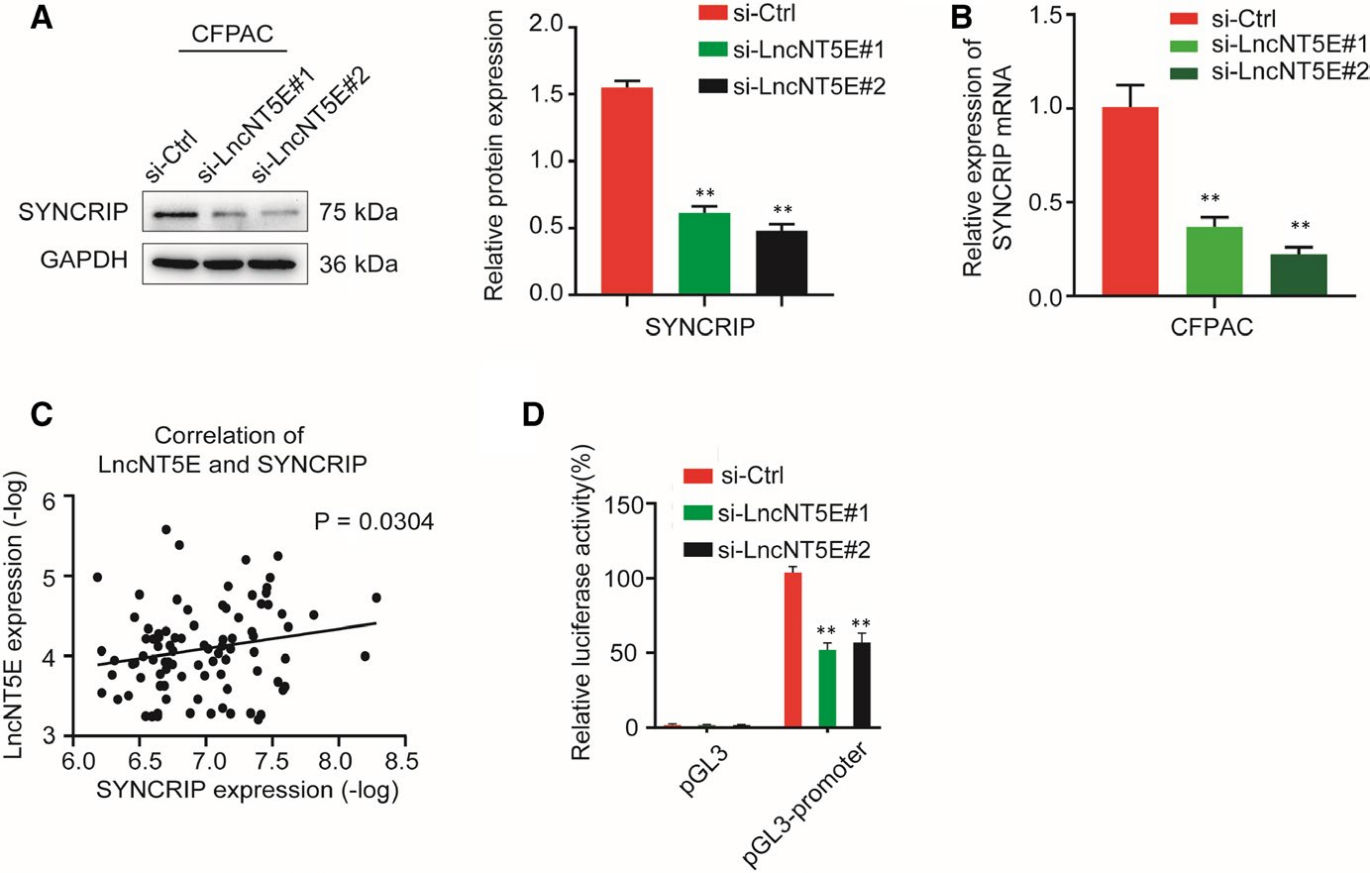
LncNT5E promotes transcription of SYNCRIP. A and B, Effect of lncNT5E depletion on the expression level of SYNCRIP in CFPAC cells, as detected by Western blotting (A) and qRT‐PCR (B). C, The correlation between SYNCRIP mRNA levels and lncNT5E levels in 45 pairs of PC tissues, as detected by qRT‐PCR (*P* = .0304). D, The luciferase activity of the SYNCRIP promoter was reduced by lncNT5E knockdown in 293 T cells. **P < .01

Some lncRNAs are reported to activate the transcription of closely located genes in cis^30^, ^31^, ^32^ by binding to the promoter. By using microarray results combined with bioinformatics analysis, we identified that the neighbouring coding gene of lncNT5E is synaptotagmin‐binding cytoplasmic RNA‐interacting protein (SYNCRIP). We therefore investigated the effects of lncNT5E on its nearby transcript, SYNCRIP. The results showed that lncNT5E depletion down‐regulated the expression of SYNCRIP at both transcriptional and translational levels (Figure 6A and B). We further investigated the relationship of lncNT5E and SYNCRIP in 45 pairs of PC and normal tissues. Correlation analysis according to qRT‐PCR results showed that lncNT5E was positively correlated with SYNCRIP expression in pancreatic cancer tissues (Figure 6C). Next, we examined whether lncNT5E promoted SYNCRIP expression via increasing its promoter activity. We constructed a luciferase reporter containing the SYNCRIP promoter, and the results of luciferase reporter assays showed that lncNT5E knockdown significantly reduced luciferase activity (Figure 6D). These results confirm that lncNT5E promotes the activity of SYNCRIP promoter to induce its transcription.

### 3.6 SYNCRIP is overexpressed in PC and is associated with poor prognosis

**FIGURE 7:**
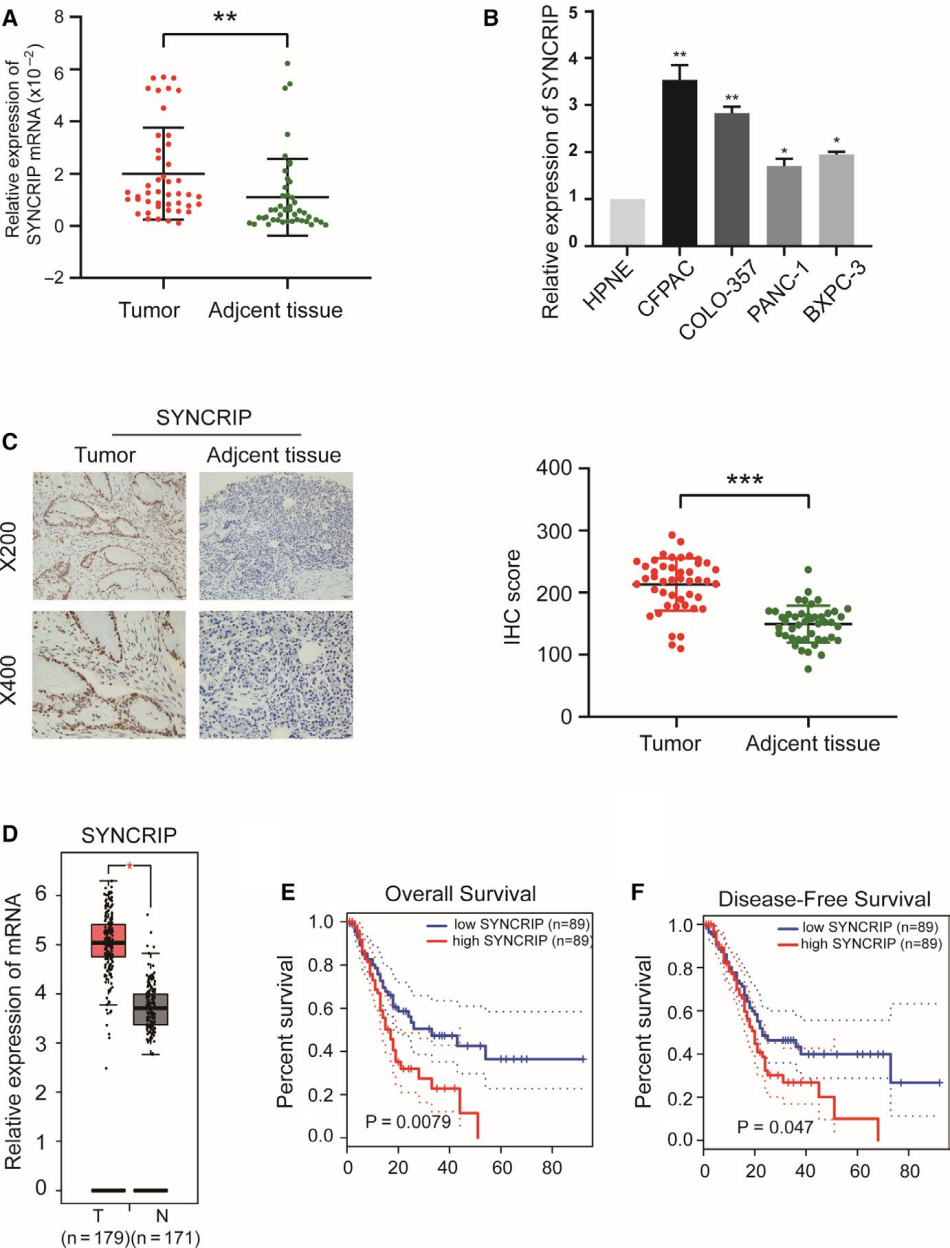
SYNCRIP is overexpressed in PC tissues and cell lines and is associated with poor prognosis. A and B, SYNCRIP expression was validated in 45 pairs of PC tissues and matched adjacent normal tissues (A) and in matched PC cells and HPNE cell lines (B) by qRT‐PCR. Data are shown as fold change (2^−ΔΔCT^). C, Immunohistochemical (IHC) staining (×200 and ×400) and H‐score for SYNCRIP in paraffin‐embedded PC and matched adjacent normal tissues. The H‐score is presented as the mean ± SD. D, Expression of SYNCRIP in PC evaluated by the GEPIA. E and F, Kaplan‐Meier curves of overall survival (E) and disease‐free survival (F) based on SYNCRIP expression levels. *P < .05, **P < .01, ***P < .001

We selected SYNCRIP as a candidate gene for further study. First, we detected the expression of SYNCRIP mRNA in 45 pairs of PC and normal tissues by qRT‐PCR. The results showed that SYNCRIP levels were significantly higher in PC tissues than in adjacent normal tissues (Figure 7A). Similar results were found in matched PC and HPNE cell lines (Figure 7B). Furthermore, immunohistochemistry (IHC) and H‐score showed that the positive rate of SYNCRIP was higher in PC tissues than in normal tissues (Figure 7C). In addition, we identified SYNCRIP as differentially expressed genes using Gene Expression Profiling Interactive Analysis (GEPIA, http://gepia.cancer‐pku.cn). The expression of SYNCRIP was clearly up‐regulated in PC tissues (Figure 7D). Moreover, survival analysis showed that the high expression of SYNCRIP was associated with poor overall survival (OS) (Figure 7E) and disease‐free survival (DFS) (Figure 7F). These results verify that SYNCRIP expression is significantly higher in PC tissues than normal tissues and is associated with poor prognosis.

### 3.7 SYNCRIP is involved in lncNT5E‐mediated proliferation and EMT of PC cells

**FIGURE 8:**
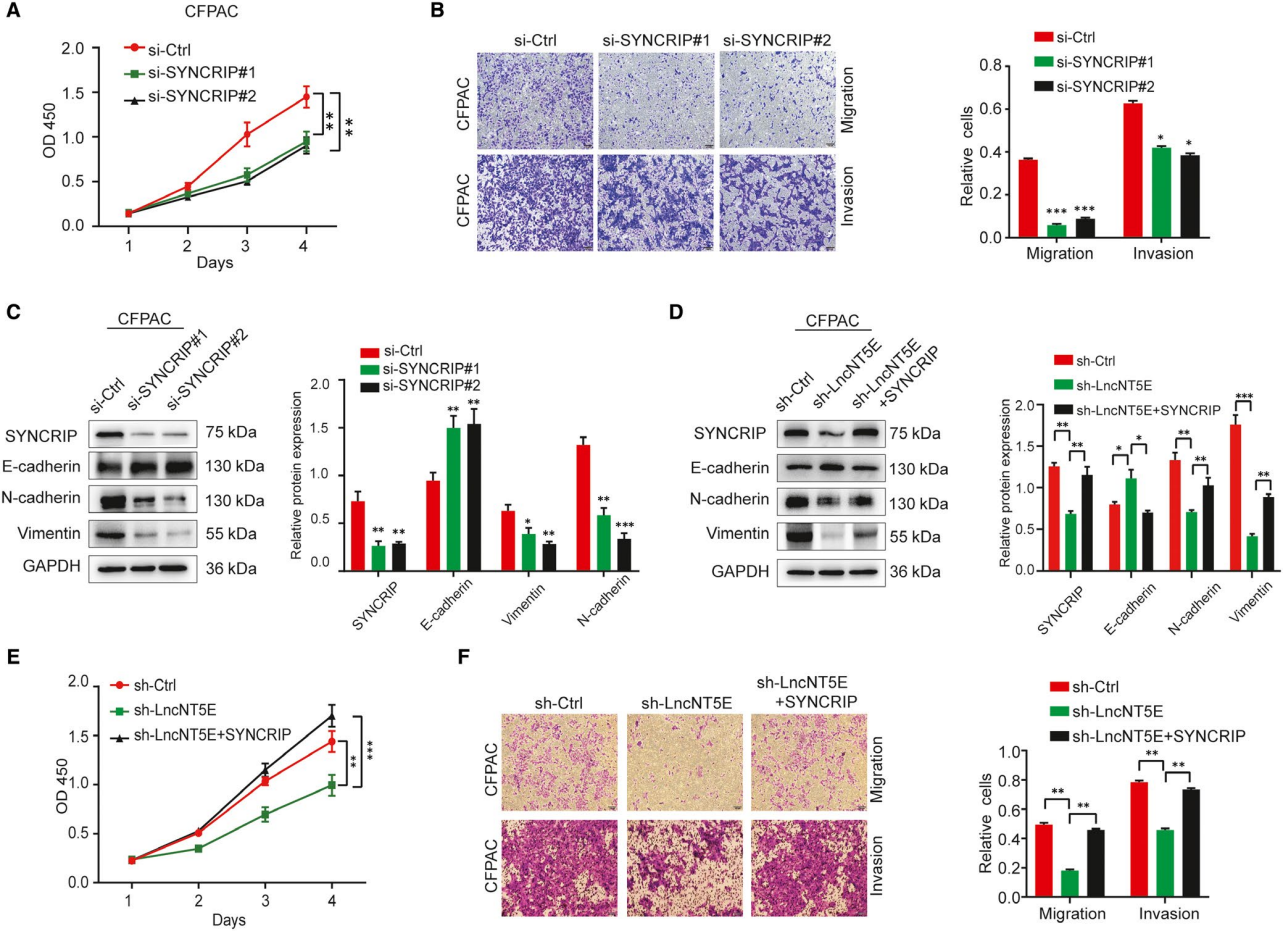
SYNCRIP is involved in lncNT5E‐mediated proliferation and EMT of PC cells. A‐C, In CFPAC cells transfected with SYNCRIP siRNA, the SYNCRIP expression level down‐regulated by si‐SYNCRIP was confirmed by Western blotting (C), the proliferation activity was determined by CCK‐8 assay (A), the migration and invasion capacity was detected by Transwell assays (B), and changes in the E‐cad, N‐cad and vimentin protein levels were verified by Western blotting (C). D‐F, Inhibition of proliferation, migration, invasion and changes in EMT‐related markers in lncNT5E‐depleted PC cells was reversed by SYNCRIP overexpression. D, The protein levels of SYNCRIP were up‐regulated, and EMT‐related marker expression was rescued after transfection with the SYNCRIP overexpression plasmid into CFPAC cells with lncNT5E depletion, as detected by Western blotting. E and F, Restoration of SYNCRIP expression significantly ameliorated the lncNT5E depletion‐induced suppression of CFPAC cell proliferation (E), invasion and migration (F). The results are shown as the protein intensity relative to that of GAPDH. Values are presented as the mean ± SD. *P < .05, **P < .01, ***P < .001

Some studies have reported that SYNCRIP expression plays an important role in cancers.^33^ We verified the function of SYNCRIP in PC cells. We confirmed that the SYNCRIP expression level was really down‐regulated by si‐SYNCRIP via Western blotting (Figure 8C). The CCK‐8 assays demonstrated that silencing SYNCRIP inhibited the proliferation of CFPAC cells (Figure 8A). Transwell assays indicated that silencing SYNCRIP inhibited PC cell invasion and migration (Figure 8B). Furthermore, SYNCRIP depletion resulted in increased levels of E‐cadherin and decreased levels of vimentin and N‐cadherin (Figure 8C).

Then, we evaluated whether the phenotypes associated with lncNT5E expression would be reversed by the restoration of SYNCRIP expression. We cotransfected the SYNCRIP overexpression plasmid into CFPAC cells with lncNT5E depletion. Overexpression of SYNCRIP was confirmed by Western blotting (Figure 8D). The CCK‐8 and Matrigel invasion assays demonstrated that restoration of SYNCRIP expression significantly ameliorated the lncNT5E depletion‐induced suppression of CFPAC cell proliferation, invasion and migration, (Figure 8E and F). Moreover, SYNCRIP overexpression rescued the increased level of E‐cadherin and decreased level of vimentin and N‐cadherin in CFPAC cells with lncNT5E depletion (Figure 8D). These findings suggest that SYNCRIP is involved in lncNT5E‐mediated proliferation and EMT in pancreatic cancer cells.

## 4 DISCUSSION

Emerging evidence has demonstrated that lncRNAs are aberrantly expressed in various cancers, including PC, and play a vital role in cancer.^34^, ^35^, ^36^ Although a large number of differentially expressed lncRNAs and mRNAs were identified between PC and corresponding adjacent normal tissues in our microarrays (H1602063, KangChen Bio‐tech Inc), only a few have been experimentally verified and functionally annotated.^26^, ^27^, ^28^ In the present study, we demonstrated that a novel antisense lncRNA, NT5E, which was identified in the microarray analysis, was markedly up‐regulated in PC tissues and cell lines. Importantly, the overexpression of lncNT5E was positively associated with TNM stage and lymph node metastasis. Moreover, the overexpression of lncNT5E was significantly correlated with a poor prognosis in PC patients, indicating that lncNT5E acted as a potential prognostic biomarker for PC. In addition, we showed that lncNT5E depletion inhibited PC cell proliferation, migration and invasion in vitro and in vivo. We also found that lncNT5E could induce EMT to promote invasion and metastasis in PC, and its nearby protein‐coding gene SYNCRIP might be involved. These results suggested that lncNT5E might be a novel therapeutic target for PC.

Pancreatic cancer is a highly malignant tumour and has become one of the leading causes of cancer‐related mortality worldwide because of the lack of early diagnosis and effective treatments. In our study, lncNT5E was initially identified to be significantly up‐regulated in PC tissues. Moreover, the high expression of lncNT5E was closely correlated with TNM stage and lymph node metastasis in PC patients. In addition, patients with high lncNT5E expression displayed worse OS and PFS. More importantly, lncNT5E was an independent prognostic factor for PC patients. Notably, perineural invasion also served as an independent risk factor for prognosis, which was consistent with the results of previous studies.^37^, ^38^ The results demonstrated that lncNT5E may play an imperative role in the tumorigenesis and progression of PC.

We further identified and explored the biological function of lncNT5E in vitro and in vivo by performing loss‐of‐function and gain‐of‐function experiments. Our results showed that the down‐regulation of lncNT5E significantly inhibited the proliferation, migration and invasion of PC cells. Moreover, lncNT5E depletion resulted in increased epithelial marker E‐cadherin and decreased mesenchymal markers vimentin and N‐cadherin, indicating that lncNT5E induced epithelial‐mesenchymal transition (EMT) reversion. In addition, the stable knockdown of lncNT5E inhibited tumour growth in vivo. These data revealed that lncNT5E exhibited strong oncogenic activity in PC.

LncRNAs have been proposed to exert diverse functions, such as transcriptional regulation in cis or trans, organization of nuclear domains and regulation of proteins or RNA molecules.^39^ Recent evidence has verified that lncRNAs can regulate local chromatin structure and/or neighbouring gene expression in cis.^30^, ^31^, ^32^, ^40^ For example, lncXLOC_000647 down‐regulates the expression of its neighbouring gene NLRP3 to suppress the progression of pancreatic cancer.^27^ Similarly, lncPCTST inhibits the progression of pancreatic cancer by down‐regulating TACC‐3.^26^ Specifically, antisense lncRNAs act as significant modulators of the expression of their neighbouring genes in cis. Therefore, we predicted that lncNT5E might promote oncogenic activity by regulating its local protein‐coding gene expression. Based on this theory, we found that SYNCRIP is a protein‐coding gene near lncNT5E. Synaptotagmin‐binding cytoplasmic RNA‐interacting protein (SYNCRIP), a member of the cellular heterogeneous nuclear ribonucleoproteins (hnRNP) family, regulates alternative splicing, polyadenylation and other aspects of mRNA metabolism and transport. A recent study confirmed that SYNCRIP is a highly conserved RNA‐binding protein with a sequence‐specific RNA‐binding domain, designated NURR (N‐terminal unit for RNA recognition), that mediates exosomal miRNA transfer, indicating an important role in the immune response, in the functioning of the nervous system and in cancer.^33^ Similarly, Vu et al reported that SYNCRIP is a new RNA‐binding protein that controls the myeloid leukaemia stem cell programme, and its depletion increases apoptosis and differentiation while delaying leukemogenesis.^41^ Moreover, SYNCRIP was confirmed to be a component of stress granules (SGs) in MDA‐MB‐231 human breast cancer and SW480 colorectal cancer cells.^42^ However, the role of SYNCRIP in PC has not been elucidated. Here, our results revealed that SYNCRIP was aberrantly highly expressed in PC tissues and cell lines. Moreover, knockdown of SYNCRIP effectively inhibited PC cell proliferation, migration and invasion and reversed EMT by increasing E‐cadherin and decreasing vimentin and N‐cadherin. Furthermore, the survival analysis showed that the high expression of SYNCRIP was related to poor clinical outcome. These data indicated the potentially essential role of SYNCRIP in promoting PC.

Importantly, we found that the SYNCRIP mRNA level was positively associated with lncNT5E expression in PC tissues. Luciferase reporter assays revealed that lncNT5E knockdown could repress the luciferase activity of the SYNCRIP promoter, which confirmed that lncNT5E might promote the activity of the SYNCRIP promoter to induce its transcription. Nevertheless, the molecular mechanism of lncNT5E regulating the activity of SYNCRIP promoter warrants further investigation. There were few literature regarding transcription factors or regulatory complexes related to SYNCRIP. A recent study reported that, based on utilizing the publicly available gene expression data set and ChIP results, the transcription factor Fubp1 can bind to the promoter region of SYNCRIP and promote its transcription.^43^ Furthermore, it was reported that Fubp1 can form an RNA‐protein complex with lncRNA.^44^ Accordingly, we speculated that lncNT5E might recruit Fubp1 to regulate SYNCRIP expression. This speculation needs more evaluation in our further experiments.

Additionally, the expression of SYNCRIP was decreased in PC cell lines upon lncNT5E down‐regulation, which confirmed our hypothesis. Moreover, the inhibition of invasion capacity in lncNT5E‐down‐regulated PC cells was rescued by SYNCRIP overexpression. Similarly, lncNT5E depletion‐induced EMT was also reversed by SYNCRIP overexpression. Thus, our data revealed the significance of the interaction between lncNT5E and SYNCRIP in PC tumorigenesis and progression, given that lncNT5E exerted its oncogenic function partly via positive regulation of SYNCRIP in PC cells.

In conclusion, our work identified a novel lncRNA, NT5E, which is up‐regulated in PC tissues and cell lines and associated with poor prognosis. The results showed that lncNT5E exerted strong oncogenic functions by promoting PC cell proliferation, migration and invasion both in vitro and in vivo. LncNT5E accelerated EMT and promoted the progression of PC possibly through the up‐regulation of SYNCRIP expression. Taken together, our findings provide insight into lncNT5E as a promising therapeutic target that could regulate SYNCRIP in PC.

## CONFLICT OF INTEREST

The authors declare no potential conflicts of interest.

## AUTHOR CONTRIBUTIONS

**Pengbo Zhang**: Conceptualization (Lead); Data curation (Lead); Formal analysis (Lead); Funding acquisition (Equal); Investigation (Lead); Methodology (Lead); Project administration (Lead); Resources (Equal); Software (Equal); Supervision (Equal); Validation (Lead); Visualization (Lead); Writing‐original draft (Lead); Writing‐review & editing (Lead). **Meng Cao**: Conceptualization (Equal); Data curation (Equal); Formal analysis (Equal); Investigation (Equal); Methodology (Equal); Project administration (Equal); Resources (Equal); Software (Equal); Validation (Equal); Visualization (Equal); Writing‐review & editing (Equal). **Yi Zhang**: Conceptualization (Equal); Data curation (Equal); Formal analysis (Equal); Investigation (Equal); Methodology (Equal); Project administration (Equal); Resources (Equal); Software (Equal); Supervision (Equal); Validation (Equal); Visualization (Equal). **Lei Xu**: Conceptualization (Equal); Data curation (Equal); Formal analysis (Equal); Investigation (Equal); Methodology (Equal); Project administration (Equal); Resources (Equal); Software (Equal); Supervision (Equal); Validation (Equal); Visualization (Equal). **Fanchao Meng**: Conceptualization (Equal); Data curation (Equal); Formal analysis (Equal); Investigation (Equal); Methodology (Equal); Project administration (Equal); Resources (Equal); Software (Equal); Supervision (Equal); Validation (Equal); Visualization (Equal). **Xinquan Wu**: Conceptualization (Equal); Data curation (Equal); Formal analysis (Equal); Methodology (Equal); Project administration (Equal); Resources (Equal); Software (Equal); Validation (Equal); Visualization (Equal). **Tianfang Xia**: Conceptualization (Equal); Data curation (Equal); Formal analysis (Equal); Investigation (Equal); Methodology (Equal); Project administration (Equal); Resources (Equal); Software (Equal); Supervision (Equal). **Qun Chen**: Conceptualization (Equal); Data curation (Equal); Formal analysis (Equal); Investigation (Equal); Methodology (Equal); Project administration (Equal); Resources (Equal); Software (Equal); Supervision (Equal). **Guodong Shi**: Conceptualization (Equal); Data curation (Equal); Formal analysis (Equal); Investigation (Equal); Methodology (Equal); Project administration (Equal); Resources (Equal); Software (Equal); Supervision (Equal). **Pengfei Wu**: Conceptualization (Equal); Data curation (Equal); Formal analysis (Equal); Investigation (Equal); Methodology (Equal); Project administration (Equal); Resources (Equal); Software (Equal); Supervision (Equal). **Lei Chen**: Conceptualization (Equal); Data curation (Equal); Formal analysis (Equal); Investigation (Equal); Methodology (Equal); Resources (Equal); Software (Equal). **Zipeng Lu**: Conceptualization (Equal); Data curation (Equal); Formal analysis (Equal); Investigation (Equal); Methodology (Equal); Resources (Equal); Supervision (Equal). **Jie Yin**: Conceptualization (Equal); Data curation (Equal); Formal analysis (Equal); Investigation (Equal); Methodology (Equal); Resources (Equal); Software (Equal); Supervision (Equal). **Baobao Cai**: Conceptualization (Equal); Data curation (Equal); Formal analysis (Equal); Methodology (Equal); Resources (Equal); Software (Equal); Supervision (Equal). **Shouji Cao**: Conceptualization (Equal); Data curation (Equal); Formal analysis (Equal); Investigation (Equal); Methodology (Equal); Resources (Equal); Software (Equal). **Yi Miao**: Conceptualization (Equal); Data curation (Equal); Formal analysis (Equal); Funding acquisition‐Supporting); Investigation (Equal); Methodology (Equal); Project administration (Equal); Resources (Equal); Software (Equal); Supervision (Equal); Validation (Equal); Visualization (Equal). **Kuirong Jiang**: Conceptualization (Equal); Data curation (Equal); Formal analysis (Equal); Funding acquisition (Lead); Investigation (Equal); Methodology (Equal); Project administration (Lead); Resources (Lead); Software (Equal); Supervision (Equal); Validation (Lead); Visualization (Equal); Writing‐original draft (Equal); Writing‐review & editing (Lead).

## Data Availability

The data that support the findings of this study are available from the corresponding author upon reasonable request.
