# Correction to “A Novel Antisense lncRNA NT5E Promotes Progression by Modulating the Expression of SYNCRIP and Predicts a Poor Prognosis in Pancreatic Cancer”

**DOI:** 10.1111/jcmm.70231

**Published:** 2024-12-26

P. Zhang, M. Cao, Y. Zhang, et al., “A Novel Antisense lncRNA NT5E Promotes Progression by Modulating the Expression of SYNCRIP and Predicts a Poor Prognosis in Pancreatic Cancer,” *J Cell Mol Med* 24, no. 18 (2020): 10898–10912, https://doi.org/10.1111/jcmm.15718.

In the article, there was an error in Figure 4B. The picture was mistakenly misplaced from group si‐LncNT5E#1 to group si‐LncNT5E#2. The correct Figure 4B is shown below.

**FIGURE 4:**
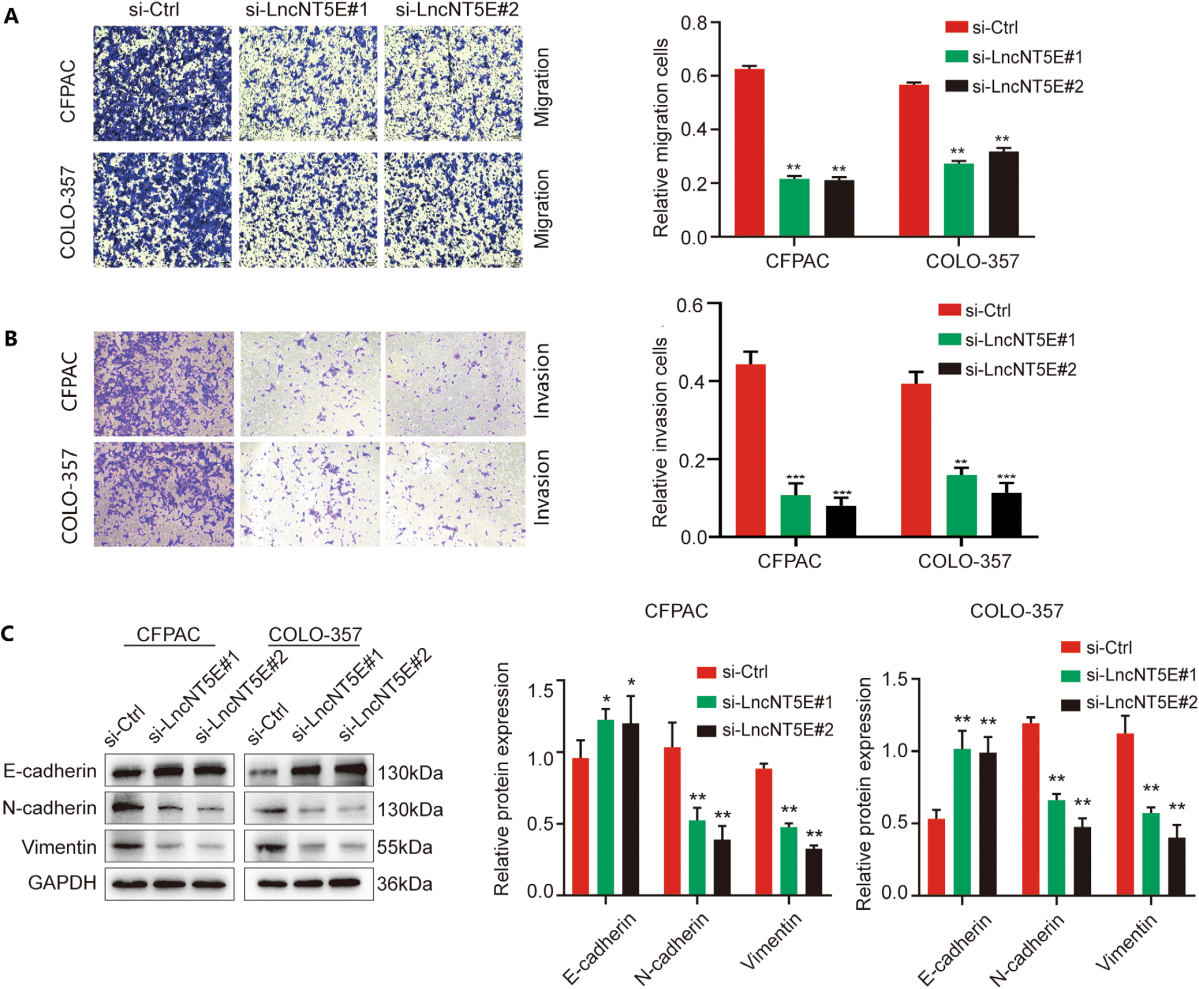
LncNT5E promotes PC cells migration, invasion and EMT in vitro. (A, B) The migration (A) and invasion (B) capacity of the CFPAC and COLO‐357 cells transfected with lncNT5E siRNA by Transwell assays. (C) Analysis of the E‐cad, N‐cad and vimentin protein levels in the CFPAC and COLO‐357 cells (si‐lncNT5E#1 or si‐lncNT5E#2 and si‐ctrl), as detected by Western blotting. Results are represented as protein intensity relative to GAPDH. *p < 0.05, **p < 0.01.

We apologize for this error.

